# Tuning Surface Chemistry of Polyetheretherketone by Gold Coating and Plasma Treatment

**DOI:** 10.1186/s11671-017-2182-x

**Published:** 2017-06-21

**Authors:** Zdeňka Novotná, Silvie Rimpelová, Petr Juřík, Martin Veselý, Zdeňka Kolská, Tomáš Hubáček, Jakub Borovec, Václav Švorčík

**Affiliations:** 1 Department of Solid State Engineering, University of Chemistry and Technology Prague, Prague, Czech Republic; 2 Department of Biochemistry and Microbiology, University of Chemistry and Technology Prague, Prague, Czech Republic; 3 Department of Organic Technology, University of Chemistry and Technology Prague, Prague, Czech Republic; 4 Faculty and Science, J. E. Purkyně University in Usti nad Labem, Usti nad Labem, Czech Republic; 5 Biology Centre CAS CR, SoWa National Research Infrastructure, Ceske Budejovice, Czech Republic

**Keywords:** Polyetheretherketone, Plasma treatment, Gold sputtering, Atomic force microscopy, Mouse embryonic fibroblasts, Scanning electron microscopy, Cell proliferation

## Abstract

Polyetheretherketone (PEEK) has good chemical and biomechanical properties that are excellent for biomedical applications. However, PEEK exhibits hydrophobic and other surface characteristics which cause limited cell adhesion. We have investigated the potential of Ar plasma treatment for the formation of a nanostructured PEEK surface in order to enhance cell adhesion. The specific aim of this study was to reveal the effect of the interface of plasma-treated and gold-coated PEEK matrices on adhesion and spreading of mouse embryonic fibroblasts. The surface characteristics (polarity, surface chemistry, and structure) before and after treatment were evaluated by various experimental techniques (gravimetry, goniometry, X-ray photoelectron spectroscopy (XPS), and electrokinetic analysis). Further, atomic force microscopy (AFM) was employed to examine PEEK surface morphology and roughness. The biological response of cells towards nanostructured PEEK was evaluated in terms of cell adhesion, spreading, and proliferation. Detailed cell morphology was evaluated by scanning electron microscopy (SEM). Compared to plasma treatment, gold coating improved PEEK wettability. The XPS method showed a decrease in the carbon concentration with increasing time of plasma treatment. Cell adhesion determined on the interface between plasma-treated and gold-coated PEEK matrices was directly proportional to the thickness of a gold layer on a sample. Our results suggest that plasma treatment in a combination with gold coating could be used in biomedical applications requiring enhanced cell adhesion.

## Background

One of the problems of human aging is wearing joints, which relate to a sharp increase in abundance of various afflictions of the skeletal and joint system including fractures, vertebral degenerations, arthritis, and bone tumors. Orthopedic surgeries using artificial implants are currently the main method used for structural and functional renewal of damaged bones and joints. Materials used commonly for orthopedic implants are especially metals, ceramics, polymers, and composites. Metal implants (e.g., gold) are widely used in the clinical practice either as permanent replacements (e.g., hip replacements, artificial teeth) or as temporal prostheses (e.g., discs, hinges, screws, and rods used to fix fractures). Metals are favored due to their mechanical strength, resistance to wear, and nontoxicity [1–3]. On the other hand, their high mechanical strength and low elasticity are incompatible with the human skeletal tissue. This might have a negative impact on a bone implant, which can lead to absorption of an adjoined bone tissue and release of the implant. Polymers such as ultra-high-molecular-weight polyethylene (UHMWPE), polytetrafluorethylene (PTFE), polymethylmethacrylate (PMMA), polylactide (PLA), polyglycolide (PGA), and polyhydroxybutyrate (PHB) are widely used in various biomedical applications. But only a limited number of polymers have been used as bone or joint replacements, since they tend to be too flexible and weak to comply with demands placed on mechanical orthopedic implants [4, 5].

Polyetheretherketone (PEEK) is a semicrystalline linear polycyclic aromatic thermoplastic polymer first synthesized in 1978 [6]. PEEK is commonly used as a material for intervertebral spacers and bone screws [7, 8]. Due to its special chemical structure, PEEK has high resistance to chemical and physical changes [6, 9, 10]; also, it is resistant to wear and is stable under high temperatures [6]. In addition, PEEK biocompatibility was proven both in vitro and in vivo and it does not cause any toxic or mutagenic effects [11–13]. Its big advantage is elasticity similar to that of a human bone, which allows balanced weight distribution between an implant and a bone; therefore, there is no stress shielding effect after implantation. However, PEEK exhibits hydrophobic and bioinert properties, which are not favorable for protein adsorption and cell adhesion [14, 15]. Thus, in order to improve these properties, the PEEK surface needs to be modified.

Surface characteristics of materials can be adjusted by various techniques [16]. One of these methods is the modification of a biopolymer surface by plasma, which uses ionized gas produced in a closed reactor system containing gas under low pressure and apparatus for electromagnetic gas excitation. Compared to wet techniques, plasma modification of a biopolymer surface is advantageous for chemical flexibility. Electromagnetically generated reactive particles interact with the biopolymer surface in a reactor causing a change in its physical and chemical properties. Mechanical, electrical, and optical properties of the material bulk, relevant to its application, remain unchanged [17], which is advantageous in the design, development, and manufacturing of biocompatible polymers. Another method used to improve polymer surface properties is cathode sputtering. Integration of gold nanoparticles into a thin film is important for various applications, for example, tissue engineering and biological sensing [18]. Nanoparticle size influences behavior and surface properties of the material surface (e.g., density, lattice parameter, electrical, and optical properties) [19]. Especially nanoparticles smaller than 100 nm often enhance cell adhesion and proliferation [20].

The goal of this work was to form nanostructures on PEEK by both plasma treatment and gold deposition to improve cell adhesion and proliferation, specifically of mouse embryonic fibroblasts (L929). The surface characteristics (polarity, surface chemistry, and structure) before and after treatment were evaluated by various techniques (gravimetry, goniometry, X-ray photoelectron spectroscopy, and electrokinetic analysis). Further, atomic force microscopy was employed to examine PEEK surface morphology and roughness. The results are discussed in the context of potential biomedical applications, mainly for possible utilization in spinal implant construction and other replacements for orthopedics and traumatology.

## Methods

### Materials and Modifications

A PEEK foil (the thickness of 50 μm, the density of 1.26 g cm^−3^, supplied by Goodfellow Ltd., UK) was used for all experiments. All PEEK samples (circular, *ø* = 2 cm) were treated by plasma which was followed by gold coating half of each sample. PEEK samples were treated in direct (glow, diode) Ar^+^ plasma using Balzers SCD 050 device (BalTec AG, Pfäffikon, CH) under conditions described in [18, 21]. Treatment times were 60 and 240 s, and the discharge power was 8.3 W. The gold coating of PEEK was accomplished by Balzers SCD 050 device from a gold target (the purity of 99.95%, supplied by Safina Ltd., CZ). The deposition conditions were DC Ar^+^ plasma; gas purity of 99.995%; sputtering times of 30, 150, and 300 s, current of 40 mA (discharge power 15 W); and total Ar^+^ pressure. The electrode distance, the power density, and the average deposition rate were adjusted similarly as described in [18, 22]. The prepared samples were stored at laboratory conditions (24 °C, 40–60% humidity) [23].

### Measurement Techniques

#### Gravimetry

The mean thickness of gold films was measured by gravimetry using Mettler Toledo UMX2 microbalance. The thickness was calculated from the sample weights before and after sputtering using the bulk density of gold. Ten samples of each type of modification were used for the measurement. The error of the gravimetric measurement was below 15%.

#### Contact Angle

Wettability of the samples was determined by the measuring of their surface water contact angles (WCA). Further, characterization of structural and compositional changes caused by plasma treatment and gold deposition was determined by Drop Shape Analysis System DSA 100 (KRÜSS GmbH, DE) at room temperature (24 °C, 40–60% humidity) [23]. Water drops of 2.0 ± 0.2 μL were deposited on the tested samples using a stainless steel needle. Images of the drops were taken after a 2-s delay. Then, the contact angles were evaluated using the ADVANCE System. At least seven measurements of different positions on at least three replicates of each sample were performed and averaged to yield the final WCA and its standard deviation. The measurement of WCA was performed on samples “aged” for 14 days.

#### X-ray Photoelectron Spectroscopy

The chemical composition of the prepared samples was determined from X-ray photoelectron spectra (XPS) measured (three measurements) by Omicron Nanotechnology ESCAProbeP spectrometer (supplied by the Omicron Nanotechnology GmbH, DE) with the relative error of 10%. The dimension of exposed and analyzed area was of 2 × 3 mm^2^. The measuring conditions were described in [18, 21]. The characteristic carbon (1*s*), oxygen (1*s*), and gold (4*f*) peaks were searched. Measuring was performed in an ultra-light vacuum. The evaluation of acquired spectra was carried out by CasaXPS code [24]. The samples used for measurement were “aged” for 14 days. Before the measurement, the samples were stored under standard laboratory conditions.

#### Zeta Potential

Electrokinetic analysis (electrokinetic potential, zeta potential) of all samples was determined by SurPASS Instrument (Anton Paar). The samples were studied inside an adjustable gap cell in the contact with an electrolyte (0.001 mol L^−1^ KCl) as well as in a buffered solution (phosphate-buffered saline (PBS)). For each measurement, a pair of polymer films with the same top layer was fixed on two sample holders (with a cross section of 20 × 10 mm^2^ and a gap between them of 100 μm). All samples were prepared in two replicates; all of them were measured three times at the constant pH of 6.8 with the experimental error of 5%. For determination of the zeta potential, the streaming current method was used and the Helmholtz–Smoluchowski equation was applied to calculate the zeta potential [25–27]. Aged samples used for measurement of the zeta potential were “aged” for 14 days.

#### Atomic Force Microscopy

Surface morphology of the samples was examined by atomic force microscopy (AFM) using VEECO CP II system (Bruker Corporation, Billerica, MA, USA). The surface was measured in a “tapping mode” using silicon P-doped probe RTESPA-CP with the spring constant of 20–80 N m^−1^ (Bruker Corporation, Billerica, MA, USA). By repeated measurements of the same region (1 × 1 μm^2^), we verified that the surface morphology did not change after three consecutive scans. The samples used for the measurement were aged for 14 days.

#### Inductively Coupled Plasma Mass Spectroscopy

Inductively coupled plasma with mass spectroscopy detector (ICP-MS) was used to determine the amount of Au ions released into PBS (pH = 7.4). The trace element analysis of Au leachates was conducted by using Agilent 8800 triple quadrupole spectrometer (Agilent Technologies, Japan) connected to an auto-sampler. Sample nebulization was performed using a MicroMist device equipped with a peristaltic pump. The uncertainty of the measurement (triplicates of each sample) was less than 3%. The leaches for ICP-MS were prepared by static incubation of the samples in PBS in humidified atmosphere with 5% CO_2_ at 37 °C for 6, 24, and 72 h. The leaches were diluted with distilled water in the ratio of 1:8 and analyzed.

### Cell Culture

According to the international standard EN ISO 10993-5, cytocompatibility testing was performed in vitro using the L929 cell line of mouse fibroblasts (Sigma, USA). PEEK samples (pristine, plasma treated, and gold coated) were sterilized in 70% ethanol in scintillation counter vials for 20 min, inserted into 12-well plates (Jet Biofil, Ø 2.14 cm), washed by PBS, and mounted onto the well bottom with hollow plastic cylinders from poly(methyl methacrylate). L929 cells were seeded on top of the samples in the density of 30,000 cells per well in 1 mL of high glucose Dulbecco’s modified Eagle’s medium (DMEM, Sigma, USA) containing 10% fetal bovine serum (FBS, Invitrogen, USA) and 2 mM stable l-glutamine (l-alanyl-l-glutamine, Sigma, USA). L929 cells were maintained at 37 °C in humidified atmosphere with 5% CO_2_.

#### Fluorescence Microscopy

After the desired incubation time (6, 24, and 72 h), the cells were fixed and stained similarly as described in [28, 29]. L929 cells were washed with PBS and fixed with 4% formaldehyde (Thermo Scientific, USA) in PBS (37 °C, 20 min). After PBS washing, F-actin of the cell cytoskeleton was labeled with phalloidin-Atto 565 (Sigma, USA) in PBS for 20 min. Then, the cell nuclei were stained with DAPI (4′,6-diaminido-2-phenylindole dihydrochloride, Sigma, USA) for 10 min, and the cells were rinsed with PBS, covered with mounting medium (Vector Laboratories, USA), and mounted between a microscopic glass slide and a coverslip. All samples (“aged” for 14 days) were tested in triplicates.

#### Scanning Electron Microscopy

Detailed morphology of the examined cells growing on pristine PEEK, plasma/gold interface, and a control (glass coverslip) was characterized by scanning electron microscopy (SEM) TESCAN LYRA3 GMU (Tescan, CZ) in a secondary electron mode. The cells intended for analysis by SEM were washed with PBS, fixed by Karnovsky solution [30, 31] in 0.1 M cacodylate buffer (pH 7.2), and dehydrated (increasing percentage of ethanol followed by two final steps of 10-min incubation in hexamethyldisilazane and drying in an oven at 40 °C for 2 h). The dehydrated samples were coated by a 10-nm gold layer.

## Results and Discussion

All measurements were performed using “aged” samples 14 days after plasma treatment and gold sputtering. It is well known that functional groups formed on a plasma-treated polymer surface are not stable and change with time [32]. The material surface tends to recover to its untreated state [33]. Therefore, it occurs to change the reorientation of the chemical groups produced by the plasma treatment into the bulk of the material [34, 35].

The PEEK ablation during the plasma treatment followed by Au sputtering was studied by gravimetry. The mass loss of a polymer caused by ablation and mass growth by sputtering were consecutively converted to polymer thickness. The mass losses determined after plasma treatment (60 and 240 s, the power of 8.3 W) are shown in Table 1. Pronounced ablation loss was apparent with increasing exposure time, but it was just doubled. The mild loss was caused probably by the aromatic character of PEEK, which resulted in enhanced resistance to cleavage than in the case of, e.g., aliphatic chains of polyolefins (UHMWPE). For gold sputtering, the time periods of 30 and 300 s were determined as representative examples of discontinuous and continuous layers [19, 36]. Decreased ablation losses have resulted in improved anchorage of gold on PEEK surface, as it is obvious for samples with 150- and 300-s gold coating.

**Table 1:** Dependence of the thickness of an ablated polymer layer on plasma exposure time (60 and 240 s) for plasma-treated and subsequently gold-sputtered PEEK (the deposition time of 30, 150, and 300 s). The change of the PEEK/plasma sample thickness was determined in contrast to pristine PEEK, the gold-sputtered PEEK in contrast to the plasma-treated sample

Sample	Ablated PEEK layer h [nm]	
	Plasma treatment [s]	
	60	240
PEEK/plasma	−29.37 ± 1.82	−68.28 ± 2.09
PEEK/plasma/30 s Au	5.68 ± 0.27	5.44 ± 0.26
PEEK/plasma/150 s Au	60.18 ± 1.59	31.23 ± 0.25
PEEK/plasma/300 s Au	119.52 ± 2.21	62.13 ± 0.46

Water contact angle values of samples measured in dependence on plasma treatment and gold sputtering time are shown in Table 2. After plasma treatment, the WCA increased from 79.5 ± 2.4° (unmodified pristine PEEK) to 94.0 ± 5.5° and to 95.6 ± 2.1° (PEEK treated by plasma for 60 and 240 s, respectively). The difference in WCA values after the plasma treatment is negligible compared to the value’s deviations. The WCA decreases with Au coating, and the surface becomes more hydrophilic compared to that in the plasma-treated samples.

**Table 2:** Dependence of water contact angle (WCA, measured by goniometry) and concentrations of carbon, oxygen, and gold (by XPS) on PEEK modification: Ar plasma-treated (8.3 W, 60 and 240 s) and Au-sputtered (30, 150, and 300 s). The error of XPS measurement was ±5%

Sample	WCA [°]	Element concentration [at.%]		
		C (1*s*)	O (1*s*)	Au (4*f*)
PEEK	79.5 ± 2.4	88.8	11.2	–
PEEK/60 s	94.0 ± 5.5	73.2	26.8	–
PEEK/60 s/30 s Au	85.3 ± 1.2	43.0	6.6	50.4
PEEK/60 s/150 s Au	85.0 ± 2.5	39.6	5.3	55.1
PEEK/60 s/300 s Au	83.2 ± 1.7	35.2	5.3	59.5
PEEK/240 s	95.6 ± 2.1	60.1	39.9	–
PEEK/240 s/30 s Au	86.0 ± 4.1	43.8	5.1	51.1
PEEK/240 s/150 s Au	82.4 ± 6.0	43.7	2.9	53.4
PEEK/240 s/300 s Au	79.8 ± 7.6	40.4	4.8	54.8

The element concentration on the polymer surface (the accessible depth of six to eight atomic layers) was examined by XPS method; the results are summarized in Table 2. The XPS data were obtained for pristine PEEK, a plasma-treated sample and a plasma-treated sample followed by Au coating. From the XPS measurement, one can see that the oxygen concentration has increased with prolonged treatment. This was probably caused by reorientation of oxygen-containing groups into the polymer volume [37–39]. It was proved that the orientation of the groups occurs immediately after the plasma treatment; thus, in the “aging process” of the sample, changes occur in the surface thereof. For this reason, the samples were measured for 14 days after the plasma treatment, when the state of “aging” of a sample is stabilized [40, 41]. The oxygen concentration increased with prolonged plasma treatment. The polymer surface is disrupted by stronger plasma discharge with creation of radical sites on the surface; the higher the plasma power, the more pronounced the polymer modification. These sites react with oxygen present in the air and increasing oxygen concentration on the treated surface [42, 43]. After gold sputtering, the concentration of oxygen decreases at the expense of the gold layer. Concentration of the gold was increased with lower sputtering time, when the surface was not so much disrupted.

**Fig. 1:**
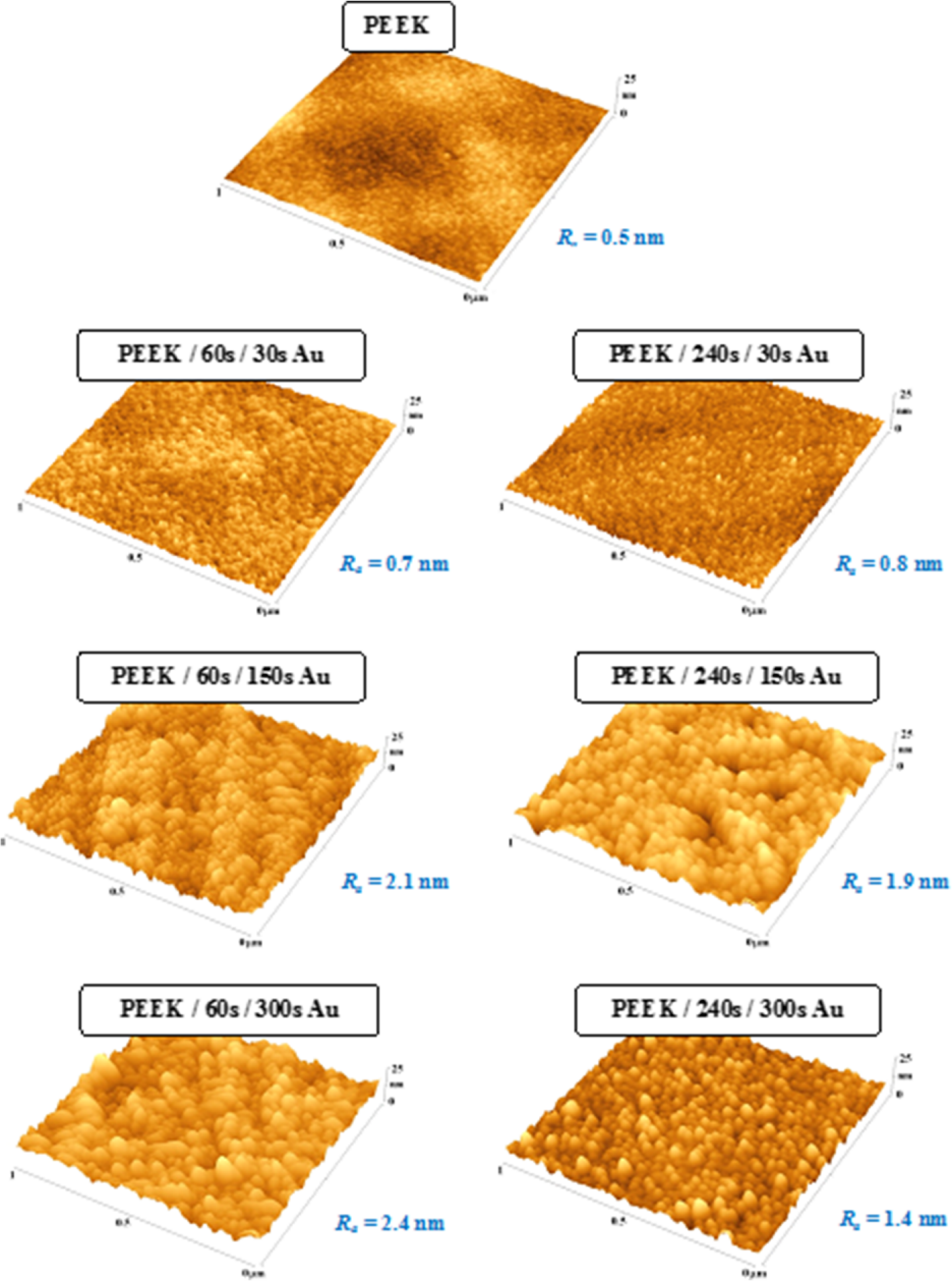
AFM images of pristine PEEK, PEEK treated by plasma (pl) for 60 and 240 s, and gold (Au) sputtered for 30, 150, and 300 s

Figure 1 shows PEEK surface morphology obtained by means of AFM. Plasma treatment caused detectable changes on the PEEK surface for both exposure times (60 and 240 s), but as expected, longer exposure to plasma resulted in a rougher surface which caused a difference in metallization of the samples. Samples treated by plasma for longer times formed better defined metal clusters. This effect was especially evident on samples with thick (sputtered for 300 s) and also very thin (sputtered for 30 s) gold layers. Samples treated by plasma for a short period (60 s) formed smaller number of larger and irregular clusters. As for layers sputtered only for 30 s, metal clusters were easily recognizable only on the substrate treated by plasma for 240 s. As expected, cluster size and surface roughness generally increase with prolonged time of gold sputtering. This behavior corresponds well with XPS measurement. Samples sputtered with metal for 30 s had already most of their surface covered by metal and further sputtering caused mostly vertical growth of clusters; therefore, it did not have such a significant impact on the gold concentration and still left a partially uncovered polymer surface. Also, a difference in a shape of the clusters in dependence on the length of plasma treatment corresponded well with XPS findings. Irregular large clusters on samples treated by plasma only for a short period (60 s) covered relatively large portion of PEEK surface; therefore, gold concentration determined by means of XPS was slightly increased. Morphological differences between pristine materials sputtered with a metal layer of different thickness can be compared to data obtained for poly-l-lactic acid (PLLA) [44] and polytetrafluorethylene (PTFE) [45]. Pristine PTFE has a very rough surface; therefore, we did not observe formation of small metal clusters but a general decrease in surface roughness. This is caused by a phenomenon that a sputtered metal prefers to fill “valleys” on the polymer surface to remain on “peaks.” On the other hand, the PLLA surface shows increased granulation and formation of similar structures to structures on PEEK, but with lower regularity. These data suggests that formation of a regular granular structure on sputtered polymers is heavily influenced by regularity of the polymer surface; therefore, PEEK (polymer with the smallest surface roughness among PEEK, PLLA, and PTFE) allows formation of most regular metal clusters on the surface.

**Fig. 2:**
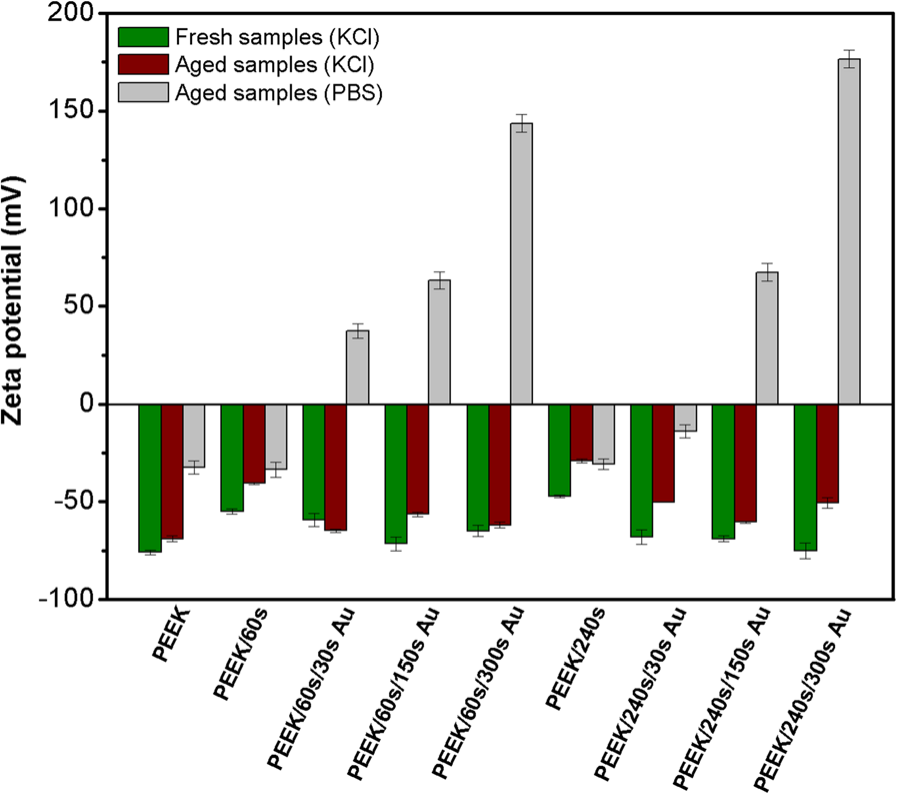
Zeta potential of plasma-treated PEEK (60 and 240 s) and Au-sputtered (30, 150, and 300 s; current 40 mA) PEEK samples in 1 mM KCl solution (*green columns*—fresh samples; *brown columns*—aged samples) and in PBS solution (*gray columns*)

Electrokinetic analysis showed changes in PEEK surface chemistry and charge after individual steps of surface modification. In the first step, plasma treatment led to creation of new polar groups on the PEEK surface which resulted in an increase in zeta potential [19, 25–27]. In the second modification, deposition of gold clusters on the sample surface had also an impact on the surface chemistry and zeta potential (Fig. 2). Due to the presence of a metal on the polymer surface, the effect of a surface charge change plays an important role—accumulation of electrons [27, 46]. We also observed different zeta potentials for fresh and “aged” samples. More dramatic changes were detected for zeta potential measured in PBS, which was given by different concentrations ions. While in KCl solution, the concentration of KCl is 0.001 mol L^−1^; in PBS, the ion concentration is three orders higher. A higher concentration of ions in PBS causes pressing of an electric double layer and results in decreased zeta potential (in absolute value) [27, 46]. Therefore, the much higher concentration of ions caused a change in the surface charge even from negative to positive values. Thus, the changes of PEEK zeta potential determined in PBS solution were more dramatic and changes in surface chemistry and charge were more pronounced. Hence, it is clear that plasma treatment and subsequent Au deposition lead to dramatic changes in polymer surface chemistry and charge that depend on the length of plasma treatment as well as on Au deposition. These results confirmed other performed analyses.

To determine the concentration of gold released into a cultivation medium during cell cultivation, we used a simple aqueous system of PBS in order to simulate these conditions. Gold released into PBS after 6 (time of cell adhesion) and 72 h (cell proliferation) of static incubation was measured by ICP-MS, and the results are summarized in Table 3. PBS has the same pH and osmolarity as the cell culture medium; thus, it was used for the ICP-MS measurement. On the one hand, PBS is a simplified system; on the other, there are no components potentially interfering with the ICP-MS measurement, as is in the case of a complete cell culture medium. We found that the concentration of Au released into PBS was higher for PEEK samples coated with Au for 30 s than that for 300 s. This could be caused by a discontinuous character of the gold layer possibly forming small clusters of gold which are dissolved faster [47]. The gold was released to a higher extent from the sample PEEK/60/300 than from PEEK/240/300 since its surface was less ablated and the gold was apparently less anchored.

**Table 3:** Concentration of Au in PBS solution after 6 and 72 h of static incubation with TCPS, pristine PEEK, and PEEK with interfaces of plasma-treated (60 and 240 s) and Au-sputtered (30 and 300 s) parts. The measurement error was ±5%

Au concentration [μg L^−1^]				
Sample [h]	PEEK/60/30 Au	PEEK/60/300 Au	PEEK/240/30 Au	PEEK/240/300 Au
6	1.07	0.22	0.91	0.20
72	3.67	1.22	3.84	0.65

**Fig. 3:**
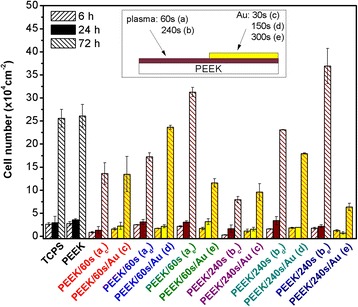
Number of L929 cells after 6, 24, and 72 h of cultivation on TCPS, pristine PEEK, and PEEK with gold interfaces of plasma-treated (60 and 240 s) and Au-sputtered (30, 150, and 300 s) regions

In the next step, it was examined whether the surface modification of polymers can promote endothelial cell adhesion. PEEK surface was activated by plasma treatment and gold sputtering. The PEEK cytocompatibility was determined based on the results of cell adhesion (6 h) and proliferation (24 and 72 h), as it is shown in Fig. 3. Each two columns (PEEK/plasma (a-b) and PEEK/(a-b)/Au (c-d-e)) represent two halves of one sample. The differences in the number of L929 cells growing on pristine PEEK and control tissue culture polystyrene (TCPS) are in the range of the measurement error. Cell adhesion was monitored 6 h after seeding of the cells on the sample surface. It is obvious that the cell number of pristine PEEK was increased when compared to that of treated samples. After 24 h of the cell growth, we observed only a very slight increase in the cell number, which might be caused by a lag phase, when the cells adapt to new environment [48]. It is apparent that 72 h after seeding, there was a very small number of cells growing on samples deposited for 30 s (60 and 240 s plasma treated) when compared with other measured samples. These values correspond with the results from ICP-MS measurements, in which gold was released into PBS to the highest extent. In this case, the Au layer deposited on PEEK (for 30 s) had a discontinuous character [36]; thus, the Au clusters could be released into the cell culture medium. By this process, the medium might become toxic for cultivated cells. The biggest cell number growing on a gold layer (compared to plasma-treated sample) was observed on the sample PEEK/pl 60 s/150 s, on which the gold layer was continuous. Next, 72 h after seeding, the most suitable environment for L929 cell growth was on the samples treated by plasma for 60 or 240 s and subsequently coated with gold for 300 s. The Au layer on these samples was also continuous [36]. According to the data from ICP-MS, only a very small amount of Au was released into the cell culture medium. However, this released gold was the likely cause of increased cell proliferation on the plasma-treated surface.

**Fig. 4:**
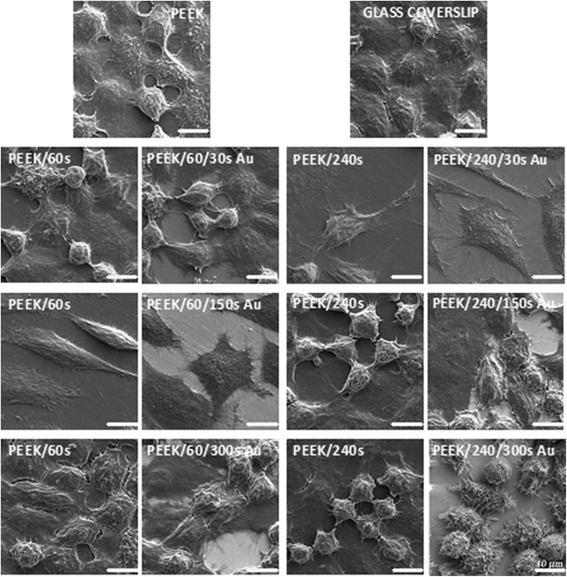
SEM images of L929 cells cultivated for 72 h on pristine PEEK, PEEK treated by plasma (60 and 240 s), and their gold-coated parts sputtered for 30 and 300 s (microscopic glass coverslip served as a control). The *scale bar* corresponds to 10 μm

To further evaluate cell morphology and intercellular connections in higher detail, we performed high-resolution scanning electron microscopy of L929 cells growing on the tested substrates; the results are shown in Fig. 4. The scans from SEM analysis were performed after 72 h of cell growth on pristine PEEK and plasma-treated and gold-coated PEEK, and a glass coverslip, which served as a control (it is commonly used for SEM analysis [49] as well as for immunofluorescence studies [29]). From Fig. 4, it is evident that cells growing on pristine PEEK, PEEK treated by plasma for 60 s (second half is 300 s Au) and for 240 s (second half is 150 and 300 s Au), and on a glass coverslip had similar shape after 72 h of cultivation. The cells were fully spread on the plasma-treated surface, and above this cell layer, formation of a new layer of proliferating cells is apparent. The cells had a spherical shape on the surface of PEEK/60 (second half is 150 s Au) and PEEK/240/30 s Au samples, even though the environment was not suitable for cell proliferation. The most rounded cells were observed on PEEK/240/300 s Au, which fully correlates with the data presented in Fig. 3.

## Conclusions

We compared two different ways of PEEK modifications in order to create a material with improved cell adhesion and growth. Obtained results confirmed variable changes in surface properties after individual modification steps. Both of employed modification ways resulted in changes in surface chemistry, morphology, wettability, and charge. The plasma treatment for 240 s caused up to two times higher weight loss of PEEK than treatment for 60 s. The wettability of the PEEK surface was not significantly changed by plasma treatment. XPS measurement confirmed the general fact that with increasing time of plasma treatment, concentration of carbon decreased in the PEEK surface, contrary to which, oxygen concentration was increased. The thickness of a deposited gold film was higher after plasma treatment for 60 s. The gold sputtering increased the surface wettability of PEEK. The results from XPS analysis showed the same trends for both plasma-treated samples (60 and 240 s), and the carbon and oxygen concentrations decreased with increasing deposition time in favor of the growing concentration of gold. AFM images also confirmed XPS measurements, especially for samples treated by plasma for 60 s and gold coated for 300 s, on which irregular large clusters covered a relatively large portion of the PEEK surface; therefore, the gold concentration was slightly increased. It was also found that samples with a thin and also a thicker gold layer are not suitable for cell propagation.

This research shows that plasma treatment improves the cytocompatibility of PEEK compared to the pristine. Also, plasma treatment is a better method for polymer modification for cell growth than gold sputtering, when gold is released into the cell culture medium.

## Abbreviations

AFM: Atomic force microscopy
CO_2_: Carbon dioxide
DAPI: 4′,6-Diaminido-2-phenylindole dihydrochloride
DMEM: Dulbecco’s modified Eagle’s medium
FBS: Fetal bovine serum
ICP-MS: Inductively coupled plasma mass spectrometry
KCl: Potassium chloride
L929: Mouse embryonic fibroblasts
PBS: Phosphate-buffered saline
PEEK: Polyetheretherketone
PGA: Polyglycolide
PHB: Polyhydroxybutyrate
PLA: Poly(l-lactide)
PMMA: Polymethylmethacrylate
PTFE: Polytetrafluorethylene
SEM: Scanning electron microscopy
TCPS: Tissue culture polystyrene
UHMWPE: Ultra-high-molecular-weight polyethylene
WCA: Water contact angle
XPS: X-ray photoelectron spectroscopy
